# Typhoon Merbok induced upwelling impact on material transport in the coastal northern South China Sea

**DOI:** 10.1371/journal.pone.0228220

**Published:** 2020-02-11

**Authors:** Chen Jiang, Ruixue Cao, Qibin Lao, Fajin Chen, Shuwen Zhang, Peiwang Bian

**Affiliations:** 1 College of Ocean and Meteorology, Guangdong Ocean University, Zhanjiang, China; 2 Guangdong Province Key Laboratory for Coastal Ocean Variation and Disaster Prediction, Guangdong Ocean University, Zhanjiang, China; 3 Marine Environmental Monitoring Centre of Beihai, State Oceanic Administration, Beihai, China; 4 Institute of Marine Science, Shantou University, Shantou, China; Guangzhou University, CHINA

## Abstract

Typhoons frequently affect the South China Sea (SCS), playing an important role in changing the coastal marine system. To determine which process has the greatest impact on material transport in the coastal marine area during a typhoon, the sea temperature, salinity, concentration of nutrients, chlorophyll-a, total suspended matter, and δ^13^C of particulate organic carbon (δ^13^C-POC) in the water column of coastal Northern SCS (NSCS) were measured during two cruises in June 2017, in the pre- and post-typhoon Merbok periods. The results show that all parameters changed significantly between the two periods. During the pre-typhoon period, stratification of nutrients and physicochemical parameters, combined to high nutrient concentrations, high temperature, and low salinity in the water column of the nearshore area, suggests that the nearshore area is influenced by the river diluted water originated in the coastal cities adjacent to our study area. In the offshore area, mineralization may be responsible for the high nutrient concentration in the bottom water. However, during the post-typhoon, the stratification of nutrients is less significant and their distribution more homogenous in the whole water column of the nearshore area. In the upper water, the nutrient concentration increased and the temperature decreased significantly. These results suggest that the enhanced vertical mixing induced by the typhoon was the dominant process in changing the nutrient distribution pattern in the coastal NSCS.

## Introduction

The South China Sea (SCS), an oligotrophic environment, is one of the largest marginal seas and fishing regions in the western Pacific Ocean, interfacing many rivers that connect it to the mainland of South China. Thus, its biogeochemistry and nutrients are very important for maintaining the ecosystem stability of this area. Recently, the input of nutrients to the coastal SCS area has greatly increased because of anthropogenic activities, causing several environmental issues, such as the formation of hypoxic areas and the acceleration of eutrophication [1–7]. However, in addition to the anthropogenic inputs from the coastal area, extreme weather events, such as typhoons and hurricanes, have been reported to influence the biological activity and geochemical cycling of nutrients in marine ecosystems [8–15].

The SCS is a region characterized by frequent typhoons. Typhoons play an important role in changing the oceanic water structure [11,13], coastal landforms, and seafloor topography [7,16,17], and in boosting phytoplankton blooms [11,18]. During a typhoon, strong cyclonic wind stress accelerates air-sea exchange and strengthens deep water upwelling and sediment resuspension [7,11,19]. Simultaneously, heavy rainfall accelerates terrigenous particle supply to the coastal areas [7,8]. These processes significantly influence the overall marine ecosystem and material transport cycles [20–22]. However, it is still unclear if the greatest impact on material transport in the coastal marine area stems from an increase in freshwater discharge or from enhanced water column mixing and deep water upwelling. In a coastal area with complex hydrological conditions, this issue needs further investigation.

To clarify this, two cruises were carried out in June 2017 during a pre- and post-typhoon, respectively, to (1) measure changes in nutrient concentrations, hydrological parameters, and δ^13^C of particulate organic carbon (δ^13^C-POC), and (2) determine the dominant process influencing nutrient transport in the coastal marine area.

## Materials and methods

### Sampling and chemical analysis

**Fig 1:**
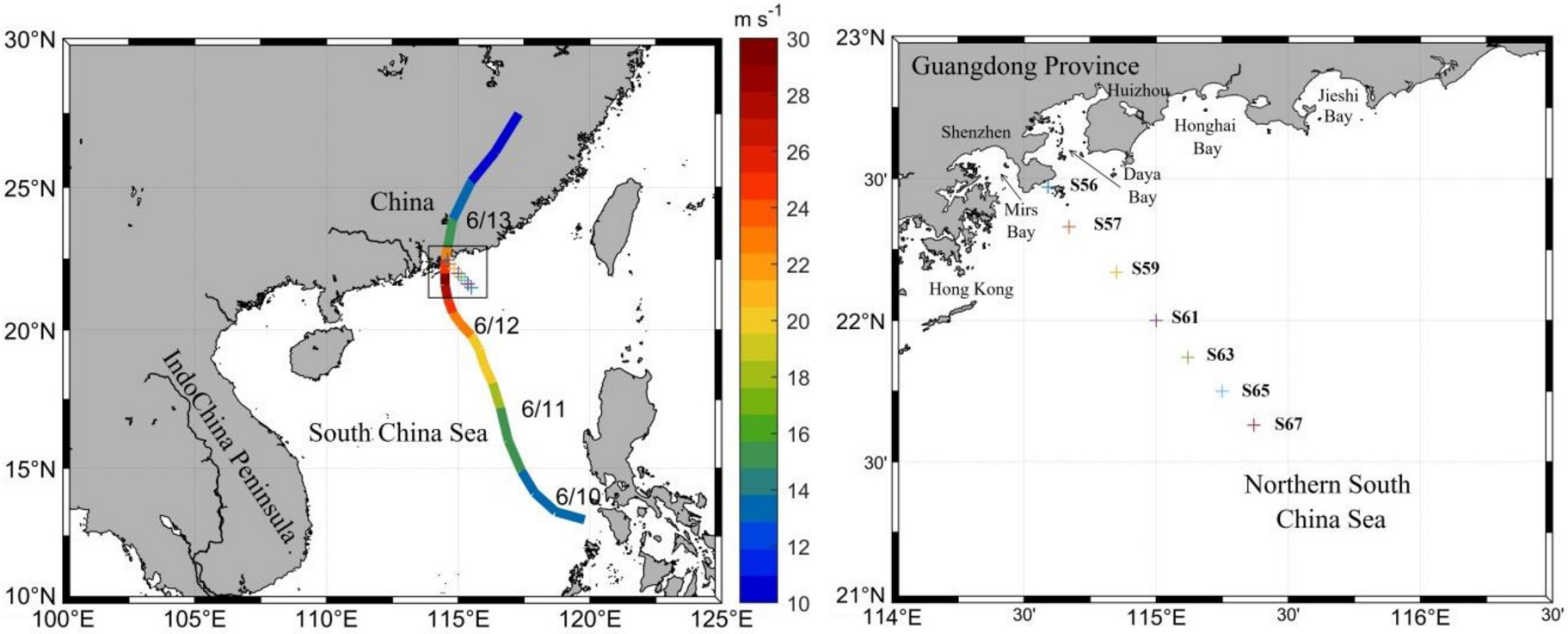
Map of the study area and track of tropical storm merbok (the black box represents the study area; the colors represent the maximum sustained wind; the plus signs represent the sampling stations). The location information: S56 (114.59°E, 22.47°N), S57 (114.67°E, 22.33°N), S59 (114.85°E, 22.17°N), S61 (115.00°E, 22.00°N), S63 (115.13°E, 21.87°N), S65 (115.25°E, 21.75°N) and S67 (115.37°E, 21.63°N).

Two sampling cruises were carried out in the summer (June) of 2017 in the coastal Northern South China Sea (NSCS), as shown in Fig 1. No specific permissions were required for the study area, because the study area did not belong to the nature reserve and not involve endangered or protected species. A total of seven sampling stations were visited at two stages, the first stage was measured on 10 June before the typhoon’s passage and the second stage was measured on 15 June during the post-typhoon period. The sampling area mainly covers the mouth of Mirs Bay, Daya Bay and the adjacent zone, and is influenced by anthropogenic activities in the adjacent cities [4,23]. Shenzhen City nearby has become a renowned metropolis in China and is rapidly developing. Its population is growing significantly because of national and international immigration. It may have a great impact on the environment of our study area. However, our study area was affected very little by the Pearl River, since the diluted Pearl River water flows along the west shore due to the Coriolis effect [4,24]. Seven sampling stations in its area were visited to investigate the spatial distribution of chlorophyll-a (Chl-a), NO_3_^-^, NO_2_^-^, SiO_3_^2-^, and PO_4_^3-^; the sampling sites are presented in Fig 1. At each station, water samples were collected by 12 L Niskin bottles at various depths (2 m, 25 m, 50 m, 75 m, 100 m) attached to a SBE911 Conductivity-Temperature-Depth (CTD) profiler measuring local temperature and salinity. Dissolved oxygen (DO) was measured onsite. For the analysis of nutrients, Chl-a, as well as for the δ^13^C-POC, water samples were filtered through precombustion (450 °C, 4 h) glass-fiber filters (47mm diameter, Whatman GF/F), and the filtrate was then stored in acid-washed polyethylene bottles at -20 °C for laboratory analysis. In the laboratory, Chl-a was extracted using 90% acetone and analysed spectrophotometrically [25], and the NO_3_^-^, NO_2_^-^, SiO_3_^2-^, and PO_4_^3-^ nutrients were determined using a San++ continuous flow analyser (Skalar, Netherlands). The data quality was monitored by intercalibration, and the detection limits for NO_2_^-^ and NO_3_^-^ were set to 0.1 μmol L^-1^. The filters for particulate organic carbon content and δ^13^C analysis were exposed to concentrated HCl vapour for at least 48 h to remove carbonates and rinsed three times with deionized water. After acidification and until analysis, the filters were freeze-dried and stored in a desiccator. All filters were packed tightly into tin cans before measuring by elemental analyzer-isotope ratio mass spectrometer (EA-IRMS) using an EA Isolink elemental analyser interfaced with a 253 Plus mass spectrometer. The reference for δ^13^C was Vienna Pee Dee Belemnite (VPDB), and the average standard deviation was ± 0.2 ‰.

### Satellite products

High-resolution satellite images were successfully captured by multi-sensors before and after the passage of Typhoon Merbok. Daily sea surface temperature (SST) data were obtained from the Microwave Optimally Interpolated (OI) SST data produced by Remote Sensing Systems (RSS), which measures SST under both clear and cloudy conditions, continuously (http://www.remss.com/measurements/sea-surface-temperature/). Because microwaves can penetrate clouds, the measurements from satellite radiometers are not influenced by cloudiness, allowing the entire area of entrainment (location of the bloom patch) to be sensed. Surface wind data are provided by the Cross-Calibrated Multi-Platform (CCMP) gridded surface vector winds, which are a Level-3 ocean vector wind analysis product by RSS (http://www.remss.com/measurements/wind/). The daily precipitation data were also derived from RSS (available online at www.remss.com/missions/amsr). The spatial resolution was 25×25 km^2^ for SST, precipitation data and 9×9 km^2^ for wind data, with a temporal resolution of 6 h. The SST and wind vector over the whole study area were available, and the daily average wind vector and speed were calculated.

### Typhoon data

Best-track data are archived in the tropical cyclone database of the China Meteorological Administration [26], available online (http://tcdata.typhoon.org.cn). The track data include maximum sustained wind, minimum sea level pressure, and the central location of storms at 6 h intervals. In our analysis, these locations were used to estimate the moving speed of typhoons. Typhoon Merbok was a fast-moving, severe storm. It originated as a tropical cyclone from the northwest of Luzon Strait on 10 June 2017, became a tropical storm on June 11 and rapidly proceeded (5.5 m/s) northwest thereafter, reaching its maximum wind speed of 30 m/s on 12 June. Then, Merbok reduced to a cyclone and made its landfall on the northeast coast of the Guangdong Province (22.5° N, 114.5° E) on 13 June (Fig 1). In the first 30 hours, the storm moved with a high speed of approximately 5.7 m/s, slowing down to 2.5 m/s on 11–12 June.

### Methodology

Ekman transport is governed by the balance of wind stress and the Coriolis force in the horizontal plane, resulting in seawater convergence and divergence at the Ekman pumping velocity. The EPV is calculated as [27],

$$EPV=curl\langle \frac{\vec{\tau }}{\rho f}\rangle ,$$ 1

where $\vec{\tau }$ is the wind stress, *ρ* = 1020 *kg m*^−3^ is the density of seawater, and *f* = 2*ω*sin *θ* is the Coriolis parameter, with *ω* the rotational velocity of the Earth, and *θ* the latitude. The wind stress *τ* is given by

$$\vec{\tau }=\rho_{a}C_{d}\left|\vec{U}\right|\vec{U},$$ 2

where *ρ*_*a*_ = 1.3 *kgm*^−3^ is the density of air, $\vec{U}$ is the wind speed at 10 m height, and *C*_*d*_ is the drag coefficient, parameterized as [28],

$$\begin{array}{llll} C_{d}\;\times \;10^{3} & = & 1.2, & |\vec{U}|\leq \;11\;m\;s^{-1}\\ & = & 0.49\;+\;0.065|\vec{U}|, & 11\;<\;|\vec{U}|\;\leq \;19\;m\;s^{-1}\;\\ & = & 1.364\;+\;0.0234|\vec{U}|-\;0.0002|\vec{U}{|}^{2}, & 19\;<\;|\vec{U}|\;\leq \;100\;m\;s^{-1} \end{array}$$

To present the spatiotemporal evolution of wind speed and wind-induced upwelling before and during Merbok, the 6 h-sea surface wind data were first processed into a daily product; then, the EPV was estimated based on these daily wind data.

## Results

### The response of the hydrological parameters

**Fig 2:**
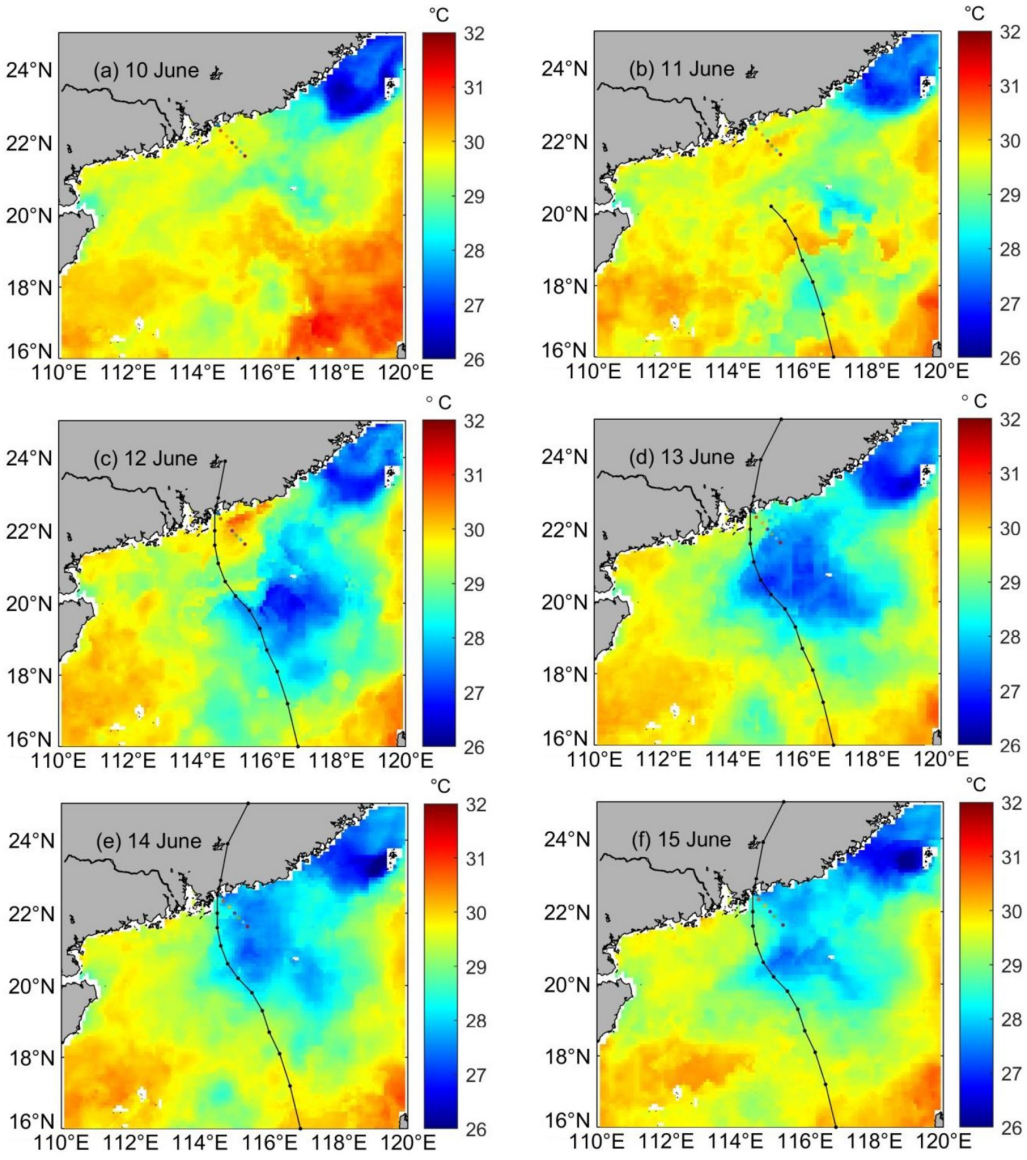
Spatial distribution map of SST in the NSCS during the passage of Merbok.

**Fig 3:**
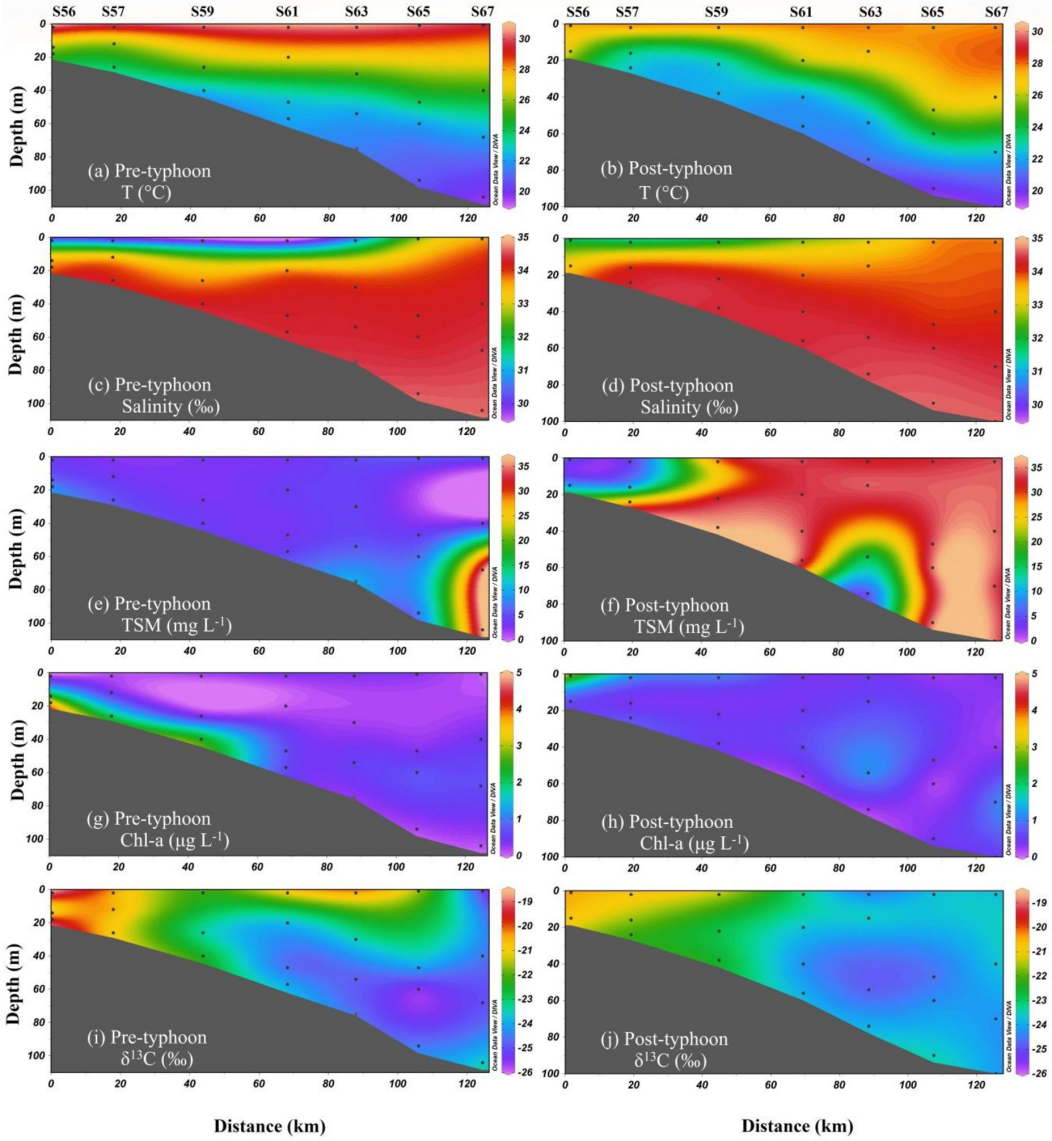
Spatial distributions of temperature, salinity, TSM, δ^13^C-POC and Chl-a on sampling sites during the pre- and post-typhoon periods.

Fig 2 shows the SST evolution during the passage of Merbok from 10 to 15 June 2017 in the north-western SCS. Before its arrival, this region was characterized by SST predominantly greater than 29 °C (Fig 2a). As Merbok passed near the sampling area, the SST dropped significantly, and a cold SST pool (26–27 °C; 115°–118° E, 19°–22° N) was observed on the track of the typhoon (Fig 2c and 2d). The minimum SST of 26.5 °C was measured at the center (117° E, 20.2° N) of the cold pool (Fig 2c). With respect to the pre-typhoon conditions (30.7 °C), the SST dropped by up to 4 °C. It should be noted that, after the passage of Merbok, the low temperature lasted for more than 3 days in the north-western SCS, where the sampling sites were located. The salinity during the post-typhoon period (ranged from 31.85 to 34.63, with an average of 33.86) was slightly higher than that pre-typhoon period (ranged from 29.52 to 34.64, with an average of 33.43). The significant change of salinity occurred in the upper water, and the salinity in the surface water increased significantly during the post-typhoon period (Fig 3c and 3d). These hydrological characteristics suggested that the low temperature and high salinity in the bottom water may be upwelling to the upper layer region due to strong mixing caused by the typhoon.

### The response of total suspended matter

The concentration of total suspended matter (TSM) was generally below 10 mg L^-1^ in the sampling sites, ranging from 2.06 to 36.28 mg L^-1^ (average of 6.53 mg L^-1^) during the pre-typhoon period (Fig 3e). It was relatively low in coastal waters, increasing gradually from the coast to the open sea as the water depth increased. The TSM concentration was significantly higher in the S67 station than in other sampling stations, and approximately 3 times higher in the post-typhoon period relative to the pre-typhoon levels (Fig 3f), ranging from 3.13 to 35.81 mg L^-1^ (average of 28.13 mg L^-1^). The distribution pattern was similar in the pre- and post-typhoon period, with high values observed in the water column of the offshore area and low values found in the upper water near the coastal area. In particular, the TSM concentration near the offshore station (S56-S57) only raised by 1.2 times, though in other stations it reached 3–10 times higher than in the pre-typhoon period. Similar to the pre-typhoon period, the TSM concentration gradually increased as the water depth increased in the post-typhoon period.

### The response of nutrients

**Fig 4:**
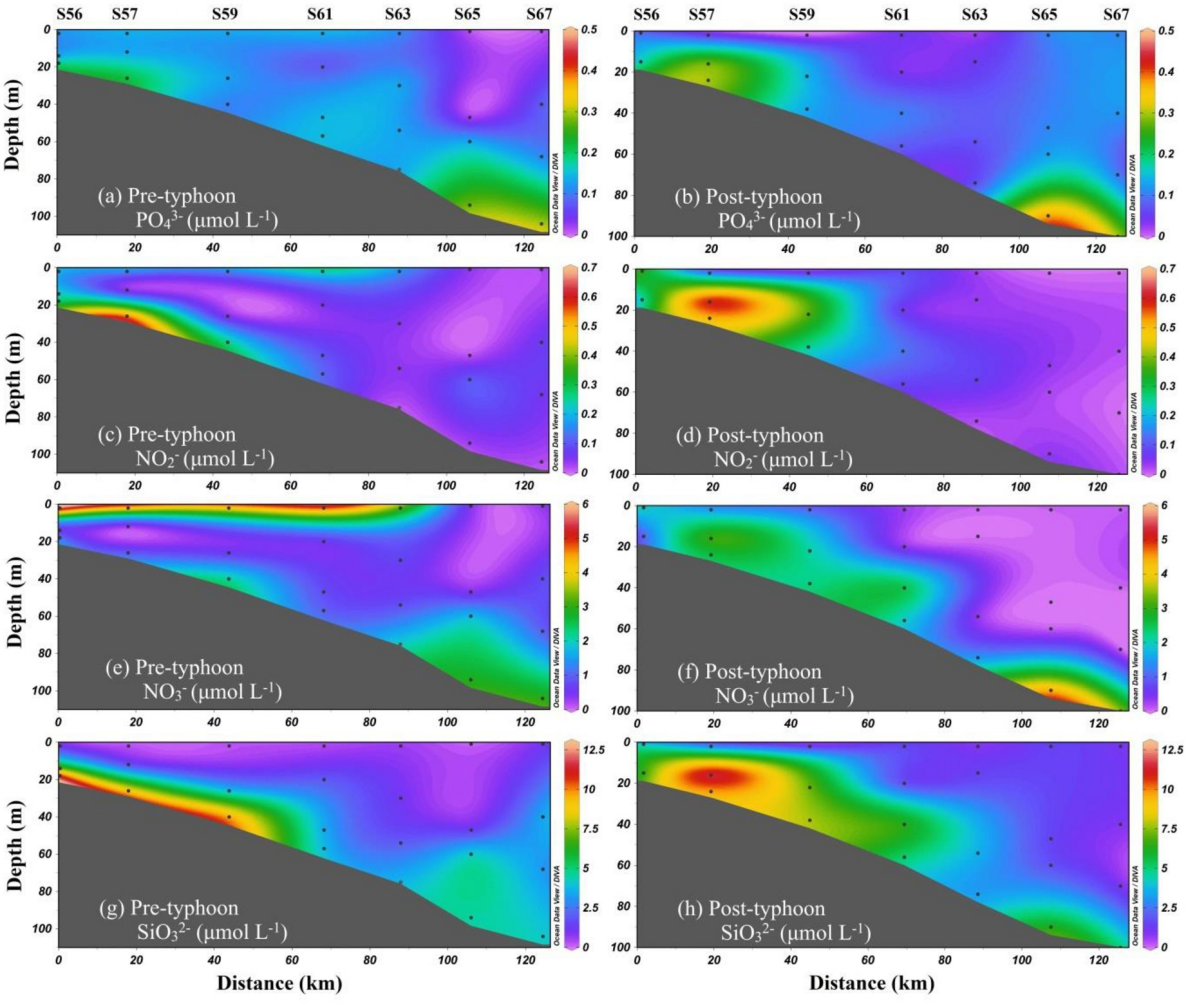
Spatial distributions of NO3-, NO2-, SiO32- and PO43- on sampling sites during the pre- and post-typhoon periods.

For Typhoon Merbok, the concentrations of NO_2_^-^, NO_3_^-^, SiO_3_^2-^, and PO_4_^3-^ in seawater changed slightly during the pre- and post-typhoon periods, as seen in Fig 4. In the pre-typhoon period, the concentrations of NO_3_^-^ and NO_2_^-^ ranged from 0.07 to 5.74 μmol L^-1^ (average of 1.78 μmol L^-1^) and from ND (no detection) to 0.58 μmol L^-1^ (average of 0.12 μmol L^-1^), respectively (Fig 4c and 4e). After the passage of Merbok, their concentrations ranged from 0.00 to 4.47 μmol L^-1^ (average of 1.28 μmol L^-1^) and from ND (no detection) to 0.64 μmol L^-1^ (average of 0.13 μmol L^-1^), respectively (Fig 4d and 4f). The distribution patterns of NO_2_^-^ and NO_3_^-^ were similar. As shown in Fig 4, after the passage of Merbok, they both exhibited higher levels in the surface water of nearshore sampling sites and lower concentrations at offshore sampling sites, implying the potential influence of terrestrial inputs. As for SiO_3_^2-^ and PO_4_^3-^, these exhibited a similar evolution at Merbok sampling sites. Before the arrival of Merbok, the concentrations of SiO_3_^2-^ and PO_4_^3-^ ranged from 0.18 to 11.18 μmol L^-1^ (average of 3.36 μmol L^-1^) and from ND to 0.30 μmol L^-1^ (average of 0.14 μmol L^-1^), respectively (Fig 4a and 4g). The distribution patterns of SiO_3_^2-^ and PO_4_^3-^ were similar. Compared with the concentrations after the passage of Merbok, the average concentrations of SiO_3_^2-^ and PO_4_^3-^ recorded in sampling sites were generally higher. After Merbok, their concentrations ranged from 0.88 to 12.41 μmol L^-1^ (mean of 3.78 μmol L^-1^) and 0.04 to 0.38 μmol L^-1^ (mean of 0.13 μmol L^-1^), respectively (Fig 4b and 4h).

### The response of δ^13^C-POC

Fig 3 shows the spatial distribution of δ^13^C-POC during pre- and post-typhoon and Table 1 shows average δ^13^C-POC. In the isotopic composition of particulate organic carbon, the δ^13^C-POC ranged from -25.7‰ to -18.9‰ (average of -23.0‰) before the passage of Merbok. High δ^13^C-POC were observed in the upper waters near the coastal areas of sampling sites, and low values were found in the water column of the offshore area. However, extremely high δ^13^C-POC were measured in the upper and mid-water column, particularly in the coastal areas. During the post-typhoon period, the δ^13^C-POC ranged from -24.8‰ to -20.4‰ (average of -23.5‰) as shown in Fig 3j. The average δ^13^C-POC in the sampling sites were slightly lower than in the pre-typhoon period. In the nearshore area, the distribution pattern of δ^13^C-POC was similar to the pre-typhoon period, though the value was generally lower, decreasing from -21.6‰ to -22.3‰. Though in the upper water column of the offshore area the δ^13^C-POC changed significantly, smaller differences were observed both in upper and mid-water columns.

**Table 1:** Average δ^13^C-POC during pre- and post-typhoon.

Station	δ^13^C_pre_ (‰)	δ^13^C_post_ (‰)	Δδ^13^C (‰)
S56	-19.8	-20.6	-0.8
S57	-20.5	-21.5	-1.0
S59	-22.7	-22.2	0.4
S61	-23.4	-23.8	-0.4
S63	-23.3	-24.1	-0.8
S65	-23.9	-24.0	-0.1
S67	-24.5	-23.9	0.6

## Discussion

### Nutrient characterization during the pre-typhoon period

During the pre-typhoon period, strong thermal stratification was observed in the sampling sites, with a high temperature in the upper water and a low temperature in the bottom water. In contrast, a high and low salinity were observed in the bottom and upper water of the coastal area, respectively. High temperature and low salinity were observed in the upper water of the nearshore area, whereas high temperature and salinity were observed in the water column of the offshore area (Fig 3a–3d). This suggests river diluted water input in the coastal area. As shown in Fig 3, all nutrients exhibited high levels in the bottom water and nearshore sampling sites, and low concentrations of SiO_3_^2-^ and PO_4_^3-^ were observed in the offshore area and upper-water column.

In agreement with the distributions of salinity and temperature, these nutrient distribution patterns suggest that the coastal area is influenced by river diluted water originated in the coastal cities adjacent to our sampling area. The sampling area mainly covers the mouth of Mirs Bay, Daya Bay and a renowned metropolis (Shenzhen City) in China. In summer, particularly, heavy rainfall resulted in greater discharges from local rivers into nearshore areas. These discharges might have carried more domestic sewage into the coastal seawater, leading to the higher nutrient concentrations observed in that season [4,9,29,30]. Furthermore, fertilizers were applied widely in the agriculture of Guangdong Province in spring and summer [5,23], and could have been carried to the coastal area by the river discharges of the latter season. Typically, marine and land plants-produced organic matter has an average δ^13^C-POC of ~ -20‰ and ~ -27‰, respectively [31,32]. Therefore, the isotopic composition of carbon can be used to distinguish algal and terrestrial contributions to POC in marine. In our study, significantly high δ^13^C-POC (average of -21.7‰, ranging from -22‰ to -19‰) and C/N ratios (average of 5.7, ranging from 5 to 8) were observed in the nearshore area, which was close to the marine sources. Accordingly, these δ^13^C-POC patterns could be resulted from the breeding and reproduction of microorganisms. We speculated that the river diluted water originated in adjacent coastal cities brought abundant water discharge and suspended particulates to the nearshore area during the rainy seasons [4,5,9,30]. Furthermore, suspend particulates are suitable for the breeding and reproduction of microorganisms, leading to intensive microbial activity and severe organic matter decomposition [33,34], resulting in relatively enriched δ^13^C-POC. This phenomenon has been observed in the coastal NSCS [35,36], and a similar result was obtained in the western North Pacific Ocean [37]. Consequently, freshwater discharge from Mirs Bay, Daya Bay causes high nutrient concentration, marine sources and decomposition result in relatively enriched δ^13^C-POC in the coastal area.

In the offshore stations, during the pre-typhoon period, high nutrient concentrations were observed in the bottom water and low concentrations in the upper water (Fig 4), combined to temperature and salinity of the upper water higher in offshore stations than in the coastal area (Fig 3a–3d). This was similar to the characteristic distribution of nutrients in the upper NSCS water [4,23]. The SCS is an oligotrophic, marginal sea in the Pacific Ocean characterized by frequent intrusion of the Kuroshio current through Luzon Strait [23,38,39]. The high temperature and salinity in the upper water of offshore stations may be influenced by the Kuroshio current, which could carry oligotrophic water into the upper SCS water [23]. In contrast, in the bottom water, high nutrient concentrations were observed during the pre-typhoon period. The reasons for this may be nitrification caused by a strong thermocline and halocline during the summer [9,23]. The study carried out by Chen et al. in the offshore NSCS showed that the ammonia concentration was significantly lower than the nitrate one in the bottom water, where dual nitrate isotopic characterization confirmed that nitrification occurred [23]. In conditions of greater nutrient availability, ^14^N and ^12^C are preferentially utilized by aquatic algae, producing isotopically lighter POC [32]. These processes may be responsible for the low δ^13^C-POC in the bottom water of the offshore area.

### The dominant upwelling process during the post-typhoon period

**Fig 5:**
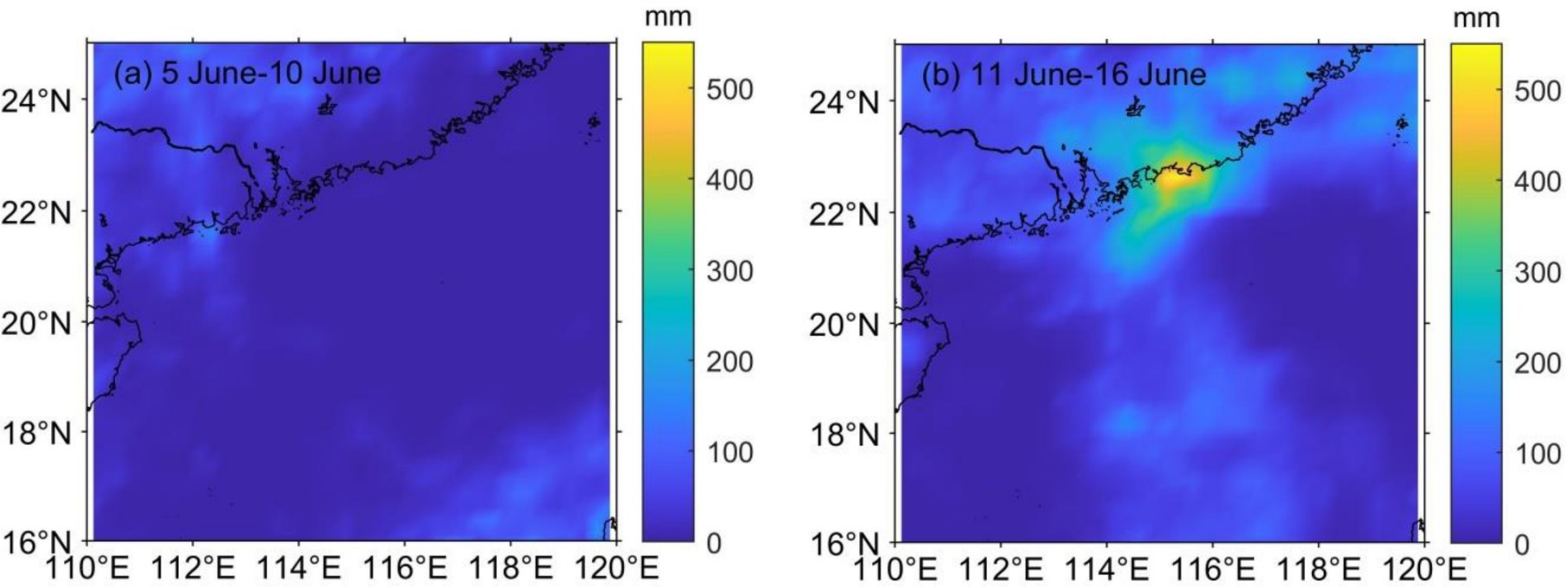
Accumulated 6-d rainfall (a) before (5 June-10 June) and (b) during and after (11 June-16 June) Merbok passage.

During the post-typhoon period, the temperature and salinity ranged from 19.63 °C to 28.13 °C (average of 24.5 °C) and from 31.84 to 34.63 (average of 33.96), respectively (Fig 3c and 3d). The temperature profile changed significantly with respect to the pre-typhoon conditions. A higher sea surface temperature of 30.42 °C was observed before Merbok passed through the sampling sites on 11 June; however, it decreased to 27.38 °C on 15 June. Simultaneously, a decrease in sea temperature was observed in the whole water column of the nearshore area. A different pattern was observed in the offshore area, with the middle water warming up slightly. After the passage of Merbok, the salinity was higher, increasing to a maximum of 1.21 at the sampling sites (Fig 3d). This is because of the cyclonic wind stress of typhoons, which can induce upwelling and bring the high salinity of subsurface seawater to the mixed layer. Then, because of stirring in the mixed layer, the high salinity water is transported to the sea surface. Additionally, the nutrient and physicochemical parameters changed significantly during the post-typhoon period, particularly in the nearshore area. The nutrients were more homogeneously distributed and their stratification decreased in the whole water column of this area, whereas their concentration increased and the temperature decreased significantly in the upper water. Two processes can result in such changes: (1) heavy rainfall during the typhoon, increasing freshwater discharge from coastal rivers (Fig 5); (2) increase of vertical mixing because of the typhoon. After the typhoon, the river runoff and freshwater discharge increase because of the heavy rainfall during its passage, and this process could carry more land-sourced pollutants into the coastal seawater [4]. However, though a slightly higher value of the δ^13^C-POC occurred in the station S59, the values generally decreased in the coastal area during the post-typhoon period. The decreased δ^13^C-POC could be explained by higher terrestrial contributions vs. decomposition of organic matters. The salinity was higher in the post-typhoon period than that in the pre-typhoon period, suggesting that the terrestrial contributions from freshwater discharge decreased in the post-typhoon period. Additionally, the increase in freshwater discharge cannot explain the decrease of 4 °C in SST. In contrast, cyclonic wind stress of typhoons can induce upwelling. With the help of continuous vertical mixing, surface sediment was turned over, and the decomposition of organic matters was promoted [40]. Thus, we speculated that terrestrial contributions should be from the surface sediment turned over, which was higher than the contributions of the decomposition of organic matters. The slightly higher value of the δ^13^C-POC may be partly influenced by the offshore movement of surface water that the low salinity surface diluted water moved offshore affected by discharged freshwater during Typhoon has shifted from S58 to S59 may result in the relatively higher value of S59. Consequently, it is concluded that Typhoon Merbok brought cold, nutrient-rich water/surface sediment up from the deep water/surface sediment to the surface layer because of strong winds and of a slow transition speed.

**Fig 6:**
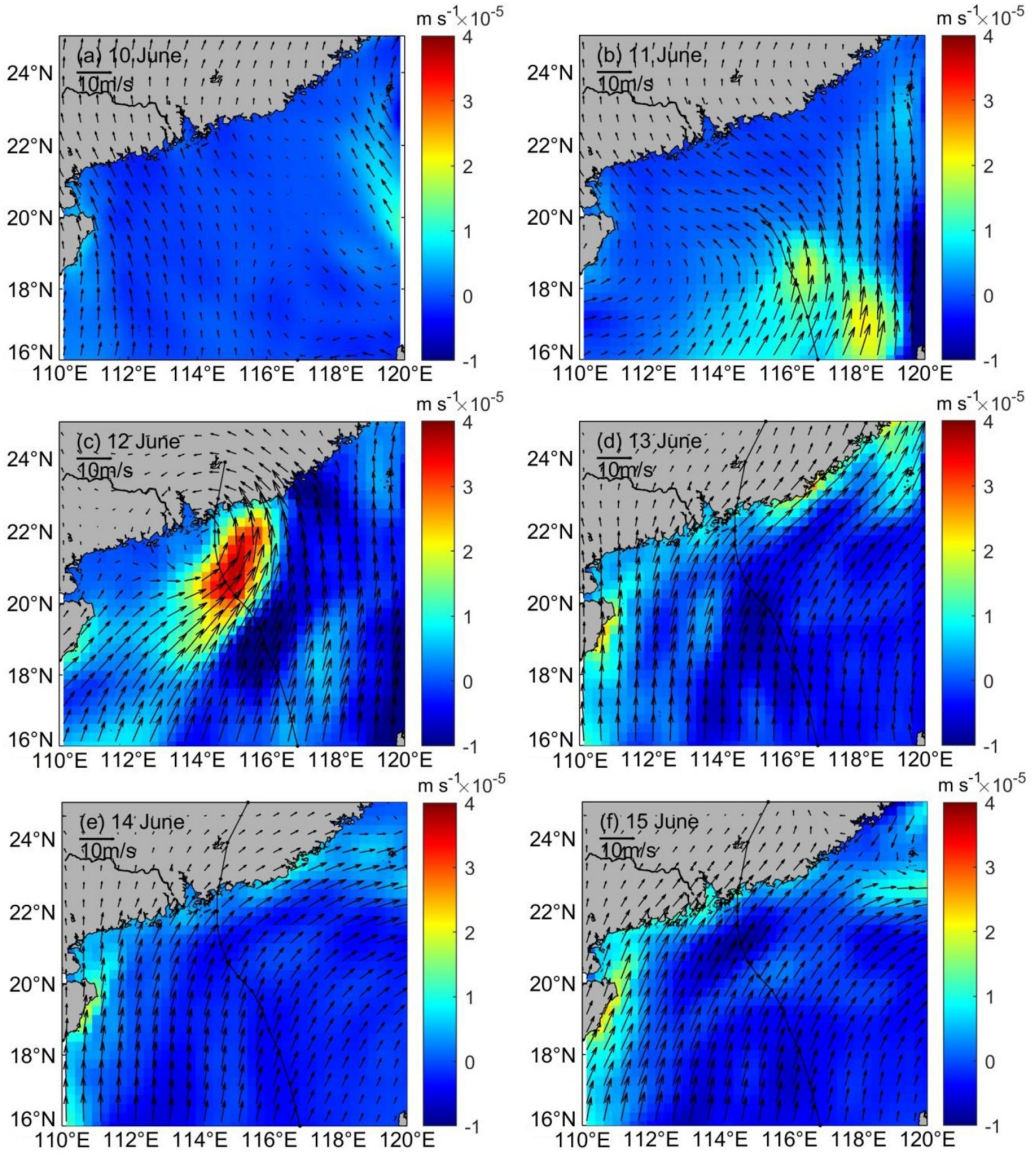
Variations of daily averaged Ekman pumping velocity in the northwestern region of NSCS before and after the storm (10 June to 15 June).

Previous studies have suggested that the sea surface temperature cooling and phytoplankton bloom during the passage of a typhoon are closely related to typhoon-induced Ekman pumping [41,42]. Here, Eqs (1) and (2) are used to calculate the value of the EPV caused by typhoons, confirming the effect of upwelling on the change of nutrient concentration. The intensity of the upwelling was significantly different on the two sides of the typhoon track. The right-hand upwelling zone was not only stronger than the left-hand side, but also larger (Fig 6c), and rightward bias occurred because, on the right side, clockwise inertial currents were accelerated as a consequence of the rotating wind stress vector of the typhoon. Merbok induced a high EPV, and the average wind speed in the study area was only threefold higher during its passage than before. However, upwelling, derived from the value of the EPV, increased significantly. The EPV reached up to 6×10^−5^ ms^−1^, which is about 2 orders of magnitude higher than the peak value in the background EPV (<1.8×10^−6^ ms^−1^, Fig 5a), near the path of Merbok. The high-EPV region (>3.1×10^−5^ ms^−1^) induced by Merbok was located near 115° E, 20.8° N, where the typhoon lingered at its slowest speed.

Under normal sea conditions, in both pre- and post-typhoon periods, the SST, TSM, and Chl-a are not significantly different in the sampling sites from the surrounding waters, and there is no sign of upwelling. However, during the typhoon, the SST significantly decreased and Chl-a significantly increased, which are typical features of upwelling. As evidenced by their daily measurements, the pre- and post-typhoon Chl-a, TSM, SST, and their distributions did not change significantly under normal sea conditions. As the impact of the typhoon increased, the Chl-a rapidly increased from 0.63 μg L^-1^ to 0.91 μg L^-1^, with especially high Chl-a concentration in the middle water column of the area, gradually decreasing towards the offshore area (Fig 3h). The TSM concentration in the coastal waters significantly increased from approximately 6.53 mg L^-1^ to 28.13 mg L^-1^. As the water depth increased, the amplitude of the TSM concentration in the water gradually decreased; however, it increased with respect to the normal sea conditions. The SST during the typhoon was lower than the normal one by approximately 2.5 °C. The region with the largest SST decrease was also the region with the largest increase in Chl-a, which is a clear sign of upwelling. During this process, the deep water is characterized by low temperature and high nutrient concentration, and resuspended particulate matter upwells to the surface water. Additionally, because the δ^13^C-POC is generally lower in the bottom water as a consequence of the degradation of organic matter [35,36], upwelling results in a decrease in the δ^13^C-POC in the upper water. Thus, compared to the increase in freshwater discharge, the increase in vertical mixing was more important during the typhoon.

## Conclusion

By comparing the SST and the distribution of nutrients, Chl-a, TSM, and δ^13^C-POC in the water column of the NSCS in a pre-typhoon and post-typhoon period, we analysed the influence of Typhoon Merbok on the NSCS marine system and determined the process of greatest impact on material transport in the coastal marine area. The results show that all parameters changed significantly between the two periods. During the pre-typhoon period, stratification of nutrients and physicochemical parameters, combined with high nutrient concentrations, high temperature, and low salinity in the water column of the nearshore area, suggests that the coastal area is influenced by the diluted water originated in the coastal cities adjacent to our study area. In the offshore area, mineralization may be responsible for the high nutrient concentration in the bottom water. However, during the post-typhoon, the stratification of nutrients is less significant and their distribution more homogenous in the whole water column of the nearshore area. Simultaneously, their concentration increased and the temperature significantly decreased in the upper water. We found that the dominant process changing the nutrient distribution pattern in the NSCS is an enhanced vertical mixing induced by the typhoon.

## Supporting information

S1 File (XLS)

S2 File (XLS)
